# Gain-of-Function STAT1 Mutation With Familial Lymphadenopathy and Hodgkin Lymphoma

**DOI:** 10.3389/fped.2019.00160

**Published:** 2019-04-30

**Authors:** Sarah E. Henrickson, Joseph G. Dolan, Lisa R. Forbes, Alexander Vargas-Hernández, Shiho Nishimura, Satoshi Okada, Leslie S. Kersun, Garrett M. Brodeur, Jennifer R. Heimall

**Affiliations:** ^1^Division of Allergy-Immunology, Children's Hospital of Philadelphia, Philadelphia, PA, United States; ^2^Institute for Immunology, Perelman School of Medicine, University of Pennsylvania, Philadelphia, PA, United States; ^3^Division of Oncology, Children's Hospital of Philadelphia, Philadelphia, PA, United States; ^4^Department of Pediatrics, Baylor College of Medicine, Houston, TX, United States; ^5^Department of Allergy, Immunology and Rheumatology, Center for Human Immunobiology, Texas Children's Hospital, Houston, TX, United States; ^6^Department of Pediatrics, Hiroshima University Graduate School of Biomedical and Health Sciences, Hiroshima, Japan

**Keywords:** STAT1, Hodgkin lymphoma, gain-of-function, primary immunodeficiency, human immunology

## Abstract

In this report, we describe a novel T437N STAT1 mutation found in a mother and 3 of her 4 children which we demonstrate yields gain-of-function. All of the four patients with the T437N STAT1 mutation experienced lymphadenopathy. However, two of the children developed Nodular Lymphocyte Predominant Hodgkin Lymphoma (NHLPL) and have responded to chemotherapeutic regimens. The fourth sibling had neither the STAT1 variant nor lymphadenopathy or malignancy. To our knowledge this is the first description of a potential association between STAT1 GOF mutations and lymphoma development.

## Background

Gain-of-function (GOF) mutations in the *STAT1* gene have been associated with a wide variety of phenotypes, including increased infection, autoimmunity and carcinoma incidence (1, 2). Genome-wide studies have shown heterozygous GOF mutations in ~50% of patients with chronic mucocutaneous candidiasis (3), which may be due to decreased levels of IL-17 and IL-22 (4, 5). Low levels of IL-17 have been independently shown to be associated with impaired immunity and manifest as increased susceptibility to fungal infections (5). Although there is an increased rate of hematologic malignancy in some primary immunodeficiency diseases (6), *STAT1-GOF* has been described to have an increased risk of carcinoma (and an overall cancer rate of nearly 6%), but it is not considered a hematologic cancer predisposition gene (1, 6).

## Case Presentations

**Figure 1:**
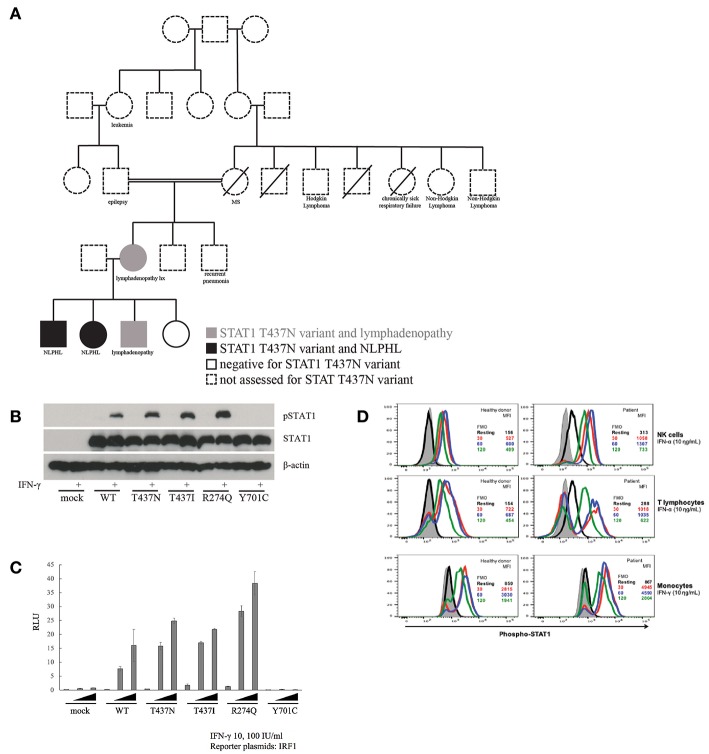
Inheritance and functional impact of STAT1 variant. **(A)** T437N family pedigree. **(B)** Immunoblot assay to assess phosphorylation of STAT1 in T437N as well as T437I (known GOF), R274Q (known GOF) and Y701C (LOF). **(C)** Transiently expressed WT or mutant STAT1 (R274Q and T437I, both GOF, and Y701C, LOF) with IRF1 reporter plasmids into STAT1 null cell line (U3C cells). Cells were stimulated with IFN-γ at varied concentrations for 16 h and IRF1 transcriptional activity was then measured with a luciferase assay. **(D)** Measurement of the effect of IFN-α and IFN-γ stimulation on the rate of STAT1 de-phosphorylation in NK cells, T cells and monocytes from patient with STAT1-T437N mutation.

The proband in this study was a boy who initially presented to the immunology clinic at age 7 with a 2-year history of persistent lymphadenopathy. There was no other history of infection, autoimmunity or malignancy. However, his mother reported that she had similar persistent adenopathy. He had a thorough clinical immune lab evaluation, including a normal number of double-negative T cells (CD4- CD8- CD3+ T cells), functional natural killer (NK) assay, and normal levels of SLAM Associated Protein (SAP, also known as SH2D1A) and X-linked Inhibitor of Apoptosis (XIAP) by flow cytometry. The only notable abnormalities on clinical laboratory studies were low CD8 T cells, advanced CD4/CD45RA:CD4/CD45RO ratio for age, and slightly low IgA and IgM (Supplemental Table 1). He was noted to have molluscum contagiosum at age 10, and was diagnosed with amplified musculoskeletal pain syndrome and panic attacks. At age 11 he continued to have molluscum and had a whole-exome sequencing (WES) performed, with a *STAT1* variant detected (1310C->A, T437N). This variant affects a conserved amino acid in the STAT1 DNA binding domain, has a CADD score of 29.1 and a minor allele frequency (MAF) of 10^−7^ using PopViz (7). Interestingly, he shares this variant with his mother and two of his three siblings (Figure 1A). No other variants that are considered likely to be pathogenic were discovered.

## Laboratory Investigations and Diagnostic Testing

**Figure 2:**
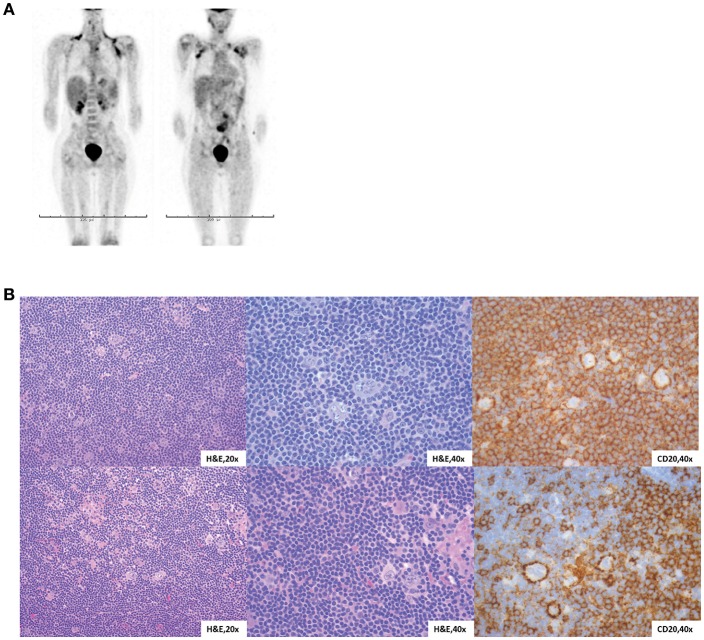
Characterization of NLPHL in STAT1 GOF patients. **(A)** Proband PET-CT at diagnosis, two views. **(B)** Lymph node biopsies from both siblings with similar morphology. H&E sections show vague nodules of small lymphocytes with sparse, large neoplastic cells with multilobulated nuclei, thin nuclear membranes, finely granulated chromatin and variable small nucleoli (“popcorn” cells).

At 12 years of age, he developed severe abdominal pain. Computerized tomography (CT) imaging demonstrated an increased volume of his abdominal lymphadenopathy as well as axillary lymphadenopathy. Subsequent biopsy of an axillary lymph node was diagnostic for Nodular Lymphocytic Predominant Hodgkin Lymphoma (NLPHL). Positron emission tomography (PET)-CT confirmed disease in the pelvis, abdomen, mediastinum and axilla (Figure 2A). Bilateral bone marrow evaluation was negative. His clinical history and imaging classified him as having Stage IIIA. Of note, he was negative for EBV by serology and PCR.

Our proband received chemotherapy with four cycles of doxorubicin, bleomycin, vincristine, etoposide, prednisone, and cyclophosphamide (AVBE-PC). After completing two cycles, staging CT scans showed >70% reduction in size of all disease sites. He received two additional cycles of AVBE-PC, and his final restaging scans showed >80% reduction of size in lymph nodes or a return of lymph nodes to a normal size. Given the response to chemotherapy, he did not receive radiation therapy (RT). A multidisciplinary decision by oncology, bone marrow transplant, and immunology was made not to perform a hematopoietic stem cell transplant (HSCT) unless there was marked clinical change, such as relapsed lymphoma or worsening immune function, due to evidence of challenging outcomes with HCT in STAT1 GOF (8).

The proband's sister (Sibling 2) presented at 5 years of age with fever, malaise and lymphadenopathy. Her constitutional symptoms resolved, but asymptomatic axillary lymphadenopathy persisted for 6–8 months. A lymph node biopsy was positive for NLPHL with no bone marrow involvement. PET-CT showed involvement of mediastinal and axillary lymph nodes without systemic symptoms and she was diagnosed with Stage IIA disease. Of note, she was also negative for EBV by serology and PCR. *STAT1* sequencing confirmed that she also carried the T437N mutation. Immunologic functional testing prior to treatment, including lymphocyte flow cytometry, immunoglobulins, and vaccine titers, was normal (Supplemental Table 1) and she does not have history of recurrent infection or autoimmunity. She shared a very similar tumor histology with her brother (Figure 2B). Given her histology and staging, she was considered to have low-risk disease and so she received three cycles of doxorubicin, vincristine, prednisone and cyclophosphamide (AV-PC). Treatment was well-tolerated, and her post-chemotherapy PET-CT showed that all involved sites had a >80% volume reduction. Due to her excellent response, there are no plans for RT or HCT.

The two affected siblings have two additional siblings who are both being followed closely clinically and have not had evidence of lymphoma (Figure 1A). Sibling 3 is a 9 year old boy who carries the familial STAT1 GOF mutation. He has ileocolic lymphadenopathy and mild (<1 cm) dilation of the appendix; serial PET CT and MRI scans have not demonstrated local or systemic disease (most recently 10 months ago) and a recent abdominal US was also stable (3 months ago). His only pathological examination was with a tonsillectomy 6 years ago and he was found to have reactive follicular hyperplasia in that tissue. He has not yet had an excisional LN biopsy and is being closely followed clinically by Immunology and Surgery with plans to follow PET every 6 months if he remains asymptomatic. The remaining sibling, Sibling 4, is a 10 year old girl who has not had a clinical history of lymphadenopathy and does not share the STAT1 variant.

To examine the impact of the T437N variant on STAT1 function, we used a number of strategies including direct study of the patient derived STAT1 variant in cell lines and direct investigation of function in primary patient cells. First, we transiently transfected STAT1-null U3C cells and measured the alteration in phosphorylation of STAT1 (pSTAT1) with IFN-γ stimulation using an immunoblot (Figure 1B). Specifically, we compared mock transfected (STAT1 negative) U3C cells, U3C cells transfected with wildtype STAT1 (WT) and U3C cells transfected with patient derived variants of STAT1. The latter included the novel T437N STAT1 variant as well as known STAT1 GOF mutations (R274Q and T437I) and a known STAT1 LOF mutation (Y701C). This demonstrated that our patient's variant led to increased pSTAT1 consistent with the known STAT1 GOF mutations. Next, we examined induction of downstream STAT1 signaling pathway cascade members (e.g., IRF1) using a luciferase assay. WT or mutant STAT1 variants were transiently expressed in the STAT1 negative U3C cell line. Transfected cells were stimulated with IFN-γ at varied concentrations for 16 h and transcriptional activity of the STAT1 downstream pathway member IRF1 was measured using a luciferase assay (Figure 1C). This demonstrated increased induction of IRF1 from T437N consistent with known STAT1 GOF variants, R274Q and T437I. Finally, we compared the rate of STAT1 dephosphorylation after IFNα and IFNγ stimulation in T437N patient vs. healthy donor NK cells, T cells and monocytes (Figure 1D). This demonstrated a delay in STAT1 dephosphorylation in the patient samples as expected in STAT1 GOF. Based on these data, we concluded that T437N is a GOF mutation in STAT1.

## Discussion

We present a case series of a single family with a novel *STAT1* GOF mutation and a clinical phenotype of lymphadenopathy and NLPHL, but no history of mucocutaneous candidiasis, other serious infection or autoimmunity. Written informed consent was obtained from the parents for publication of these cases and ongoing research based study of their children's immunodeficiency. The mother had spontaneous resolution of her enlarged abdominal nodes in early adulthood and never underwent biopsy. Three of her four children carry the same mutation, two of who were treated for Nodular Lymphocyte Predominant Hodgkin Lymphoma and the third is being closely monitored due to persistent lymphadenopathy for evidence of malignant transformation. Both of the patients with NLPHL had favorable responses to chemotherapy with minimal toxicity and neither received radiation. Of the remaining siblings, one does not carry the mutation and does not have lymphadenopathy and the other does have the mutation and is being closely clinically followed for evidence of progression or transformation of mild lymphadenopathy. So, of the known affected family members, each has either NLPHL or significant lymphadenopathy. While there are grandparents with lymphoma, unfortunately we were not able to obtain DNA for testing, so their variant status is unknown.

Patients with GOF mutations of *STAT1* have variable phenotypes reported in the literature, which include autoimmunity, increased susceptibility to infection (especially mucocutaneous candidiasis) and carcinoma (1, 9), but our patients had none of these features. The reported cancers in patients with *STAT1* GOF include oropharyngeal, esophageal, and gastrointestinal carcinomas, as well as melanoma, acute leukemia and liver cancer (1, 10, 11) There are no reported cases of Hodgkin lymphoma. Although the data are limited, the phenotype may be different depending on the site or type of mutation (genotype). HSCT has been reported for 15 of these patients, and there was 74% engraftment, 50% primary graft failure, and 40% overall survival (8).

Our report of this family with a novel mutation expands the clinical phenotype of *STAT1* GOF mutations to include lymphadenopathy and NLPHL.

## Ethics Statement

This study was carried out in accordance with the recommendations of the Children's Hospital of Philadelphia IRB with written informed consent from all subjects. All subjects gave written informed consent in accordance with the Declaration of Helsinki. The protocol was approved by the Children's Hospital of Philadelphia IRB.

## Author Contributions

SH, JD, and JH wrote the paper. LF, AV-H, SN, and SO performed experiments to analyze the effect of the familial mutation on STAT1 function. LK, GB, JD JH, and SH also cared for the family clinically. All authors reviewed and edited the paper.

### Conflict of Interest Statement

SH has served on *ad hoc* advisory boards for Horizon Pharma; unrelated to this study. The remaining authors declare that the research was conducted in the absence of any commercial or financial relationships that could be construed as a potential conflict of interest.
