# Supplementary material for: Gain-of-Function STAT1 Mutation With Familial Lymphadenopathy and Hodgkin Lymphoma
Source: Front Pediatr. 2019 Apr 30;7:160. doi: 10.3389/fped.2019.00160 (PMC6503099; doi:10.3389/fped.2019.00160)
Supplement: Supplementary file 1 [file Table_1.docx]

**Supplemental Table 1:** Presenting and most recent clinical immunologic data of all sibling carriers of STAT1 GOF T437N mutation

|  | CD3  %(cell/uL) | CD4  %(cell/uL) | CD8  %(cell/uL) | CD4/CD45RA  %(cell/uL) | CD4/CD45RO  %(cell/uL) | CD3-CD56+ &/or CD16% (cell/uL) | CD3+/CD4-/CD8-/TCRab+  %(cell/uL) | CD19  %(cell/uL) | IgG | IgA | IgM | Tetanus  titer |
| --- | --- | --- | --- | --- | --- | --- | --- | --- | --- | --- | --- | --- |
| **Proband**  Age 7y  Age 14y  (post Rx) | 87 (3075)  **81.9 high** **(1908)** | 73 (2597)  **67.1 high** **(1563)** | 12 (422)  12.7 (296) | 26 (911)  25.1 (585) | 46 (1630)  **39.9 (930) both high** | 6 (209)  6 (140) | 1 (35)  ND | 7 (248)  11.6 (270) | 683  **514 (low)** | 49  **28 (low)** | 45  **20 (low)** | 1.44  0.65 |
| **Sibling 1**  Age 5y    Age 7 (post Rx) | **82.9 high** **(2321)**  **86.0 high (2245)** | 58.7 (1644)  **54.6 high (1425)** | 21.4 (599)  26.8 (699) | ND  28.6 (746) | ND  **23.8 (621) high** | **3.6** **low** **(101)**  6.8 (177) | ND  ND | 11.9 (333)  **5.8 (151) both low** | 573  **554 (low)** | **38 (low)**  **43 (low)** | 65  **40**  **(low)** | 1.05  0.32 |
| **Sibling 2**  Age 3y  Age 5y  Age 8y  Age 9y | 57.0 (1031)  66 (1982)  73.5 (1838 )  74.6 (1775 ) | 36.4 (658)  42.7 (1282)  50.8 (1270 )  51.2 (1219 ) | **13.8 (250) low**  **16.5 (495) low**  16.4 (410)  16.8 (400 ) | 29.7 (537)  32.6 (979)  32.6 (815)  35.4 (843) | 11.5 (208)  12.1 (363)  14.4 (360)  15.6 (371) | 14.9 (270)  11.7 (351)  5.8 (145)  9.0 (214) | 1(18)  ND  ND  ND | 27.8 (503)  23 (685)  20 (500)  15.3 (364) | 628  929  606  692 | 83  95  100  142 | 93  96  70  102 | 0.58  0.23  1.0  0.88 |
